# Evaluation of long-term antinociceptive properties of stabilized hyaluronic acid preparation (NASHA) in an animal model of repetitive joint pain

**DOI:** 10.1186/ar3394

**Published:** 2011-07-07

**Authors:** Michael Karl Boettger, Diana Kümmel, Andrew Harrison, Hans-Georg Schaible

**Affiliations:** 1 Institute of Physiology I/Neurophysiology, Jena University Hospital - Friedrich Schiller University, Teichgraben 8, D-07743 Jena, Germany; 2 Bayer Schering Pharma AG, Friedrich-Ebert-Strasse 475, 42117 Wuppertal, Germany; 3 Clinical Therapies R&D, Smith & Nephew Research Centre, York Science Park, Heslington, York, YO10 5DK, UK

## Abstract

**Introduction:**

Clinical trials provided controversial results on whether the injection of hyaluronan preparations into osteoarthritic joints reduces pain. Problems of clinical studies may be the substantial placebo effects of intra-articular injections, different severity and rate of progression of the disease and others. We hypothesize that the use of preclinical pain models may help to clarify whether a certain hyaluronan exerts antinociceptive effects upon intra-articular injection. In the present study we tested in the bradykinin/prostaglandin E_2 _(PGE_2_) model primarily the putative antinociceptive effect of stabilized hyaluronic acid from a non animal source (NASHA), a stabilized hyaluronic acid based gel for intra-articular treatment of OA. We established a dose-response relationship for NASHA and we compared NASHA to other hyaluronans with different formulations that are in clinical use.

**Methods:**

To induce transient joint pain episodes bradykinin and PGE_2 _were repetitively administered intra-articularly and unilaterally into rat knee joints during short anaesthesia. After establishment of the predrug nociceptive responses, a single intra-articular injection of saline or NASHA at different concentrations was administered and pain responses to further bradykinin/PGE_2 _injections were monitored up to 56 days after NASHA. Furthermore, the obtained effective dose was compared to clinically defined concentrations of Hylan GF20 and sodium hyaluronate. The primary outcome measures were primary mechanical hyperalgesia at the knee joint and pain-induced weight bearing.

**Results:**

On day 1 after injection, all tested hyaluronan preparations showed an antinociceptive effect >50% compared to saline. Single injections of higher doses of NASHA (50, 75 and 100 μl) were antinociceptive up to 56 days. When injection volumes in rat knee joints were adapted to clinical injection volumes in humans, the antinociceptive effects of the cross-linked NASHA and Hylan GF20 had a longer duration than that of the non cross-linked sodium hyaluronate (with a slightly better effect of NASHA than Hylan GF20).

**Conclusions:**

In the bradykinin/PGE_2 _model of joint pain a single injection of all hyaluronan preparations provided significant antinociceptive effects compared to saline. It appeared that the duration of the antinociceptive effect of the cross-linked hyaluronan preparations NASHA and Hylan GF20 was more prolonged. In addition, the gel beads structure allowing only a slow release of hyaluronic acid (NASHA) may even enhance this prolonged antinociceptive effect.

## Introduction

Joint pain is among the most frequent chronic pain states [1]. In most cases, chronic joint pain results from osteoarthritis (OA), which has a prevalence of about 90% in the older population [2,3]. At this time OA cannot be cured. Therefore, symptomatic pain relief is essential because pain is one of the most disabling symptoms and can thus cause a significant aggravation of joint dysfunction [4]. Most often, nonsteroidal anti-inflammatory drugs (NSAIDs) are clinically used. NSAIDs can effectively reduce inflammation and pain, particularly in exacerbated OA [5], but can also cause significant side effects such as gastrointestinal and renal disorders [6,7] when taken regularly. Alternatively, whenever single or few joints are affected, local antinociceptive therapy might be considered. In this respect, hyaluronic acid (HA) preparations are often used. Subject to the preparation used, HA is injected into the joint one, three, or up to six times at weekly intervals [8,9]. Some studies reported good analgesic effects of HA preparations [10-13] whereas others found an antinociceptive action in the range of placebo effects [13,14]. In fact, clinical trials to prove the efficacy of HA preparations in OA are compromised by the large placebo effect in this patient group [15]. The injection of a knee is an active and invasive treatment and hence powerful placebo effects may mask true antinociceptive effects of compounds. In addition the tools to record these effects, such as Western Ontario MacMaster Questionnaire, are subjective in nature and hence a source of bias. Furthermore, it is important which patients are included. For example, one study included patients with poly-articular OA and knee effusions. For the overall population there was no significant analgesic effect but when these patients were removed from the analysis, the stabilised HA was shown to be highly efficacious over saline in patients with knee OA [13]. Comprehensive meta-analyses stressed the poor quality of many trials [16], the heterogeneity among the studies [17], and came to different conclusions, ranging from no effect [16], or a small effect, with highest-molecular-weight HA possibly being more efficacious than lower-molecular weight HA in treating knee OA [17]. The review from Bellamy et al. [18] concludes "overall, the analyses support the use of the HA class of products in the treatment of knee OA". In addition the injection of different HA preparations at different doses is usually not feasible.

In this respect, preclinical approaches may provide important background data on the antinociceptive properties of HA. For instance, in horses, intraarticular injections of HA preparations attenuated the lameness in natural and experimentally induced OA [19,20]. In anesthetized cats and rodents HA preparations reduced inflammation- and OA-induced increases of neuronal discharges in nociceptive Aδ- and C-fibres innervating the knee joint [21-24]. Herein we show an alternative preclinical approach to monitor long-term antinociceptive effects of HA preparations, namely the repetitive induction of short-lasting pain states in the joint by the injection of bradykinin, combined with prostaglandin E_2 _(PGE_2_) [25,26]. These inflammatory mediators sensitize nociceptive Aδ-and C-fibres to mechanical stimuli [27-32], a basic mechanism for the occurrence of pain upon movements in the normal working range of the joint. Firstly, we validated the described bradykinin/PGE_2 _model with regards to behavioral readout parameters in rats for a long-term study on the antinociceptive effects of stabilized hyaluronic acid from a non-animal source (NASHA) up to 56 days, and we established a dose-response relation for NASHA. Secondly, the obtained effective dose of NASHA was compared with two other clinically used preparations, that is Hylan GF20 and sodium hyaluronate, for duration and effect sizes of their antinociceptive properties.

NASHA is characterized by a gel structure which is stabilised using about 1% of cross-linking agent, thereby increasing the half-life time of the product in the joint compared with traditional HA preparations [33,34]. Thus fewer injections are necessary as compared with other compounds, which may reduce the risk of infection [5]. The efficacy of NASHA has been well documented in clinical studies [35]. Hylan GF20 is another HA product with a modified HA composition which is available as an intra-articular formulation for the treatment of OA. Here we report on the magnitude and long-term duration of antinociceptive effects of NASHA and other HA preparations in the bradykinin/PGE_2 _model of repetitive joint pain.

## Materials and methods

### Animals

Female Lewis rats (*n *= 122, age six to eight weeks, weight upon arrival 160 to 180 g) supplied by Charles River (Sulzfeld, Germany) were used. Animals were housed on a 12:12 hour light:dark cycle with water and standard rodent chow available *ad libitum*. All experiments were approved by the Thuringian state authorities (registration numbers 02-045/07 and 02-014/09) and complied with EC regulations (86/609/EEC). The Extended Methods Form for uniform reporting standards in pain-related animal experiments [36] can be found as an online supplement.

### Study design

All intra-articular injections were performed during short anesthesia with 2% isoflurane (lasting about five minutes). The assessment of pain-related and locomotor behavior was started about 30 minutes after isoflurane application when animals had fully recovered from anesthesia.

#### Validation of the bradykinin/PGE_2 _injection pain model (protocol 1, *n *= 12)

Previous models for a short-term induction of pain states employed intra-articular injections of bradykinin [21]. As such hyperalgesia lasts for minutes only, we aimed to prolong this hyperalgesia by simultaneous injection of PGE_2 _as described previously [25], which is likewise known to sensitize afferent fibers [37,38] and which is released in OA joints. For PGE_2 _(Cayman Chemical, Ann Arbor, MI, USA), a dose of 0.5 μg was used as described previously [26]. As bradykinin concentrations used in previously described models vary between 0.03 μg and 150 μg [25,26,39,40], we aimed at identifying an effective dose for our purpose, that is a dose that causes a decrease in mechanical thresholds (see below) of at least 30% lasting for at least 90 minutes. For that purpose, we chose an escalating dose design (*n *= 4 animals) using 0, 0.025, 0.075, 0.25, 0.75, 2.25, 6.75, 20.25, 60.75, and 182.25 μg of bradykinin (Sigma, Deisenhofen, Germany), diluted in saline together with PGE_2 _in a total intra-articular injection volume of 50 μl. The chosen bradykinin concentration was then verified in four bradykinin-naïve animals. In order to establish that the model indeed indicates pain-related behavior, an additional four animals were treated with morphine (2.5 mg/kg, Sigma, Deisenhofen, Germany) 30 minutes prior to injection of inflammatory mediators.

#### Dose-response relationship for NASHA (protocol 2, *n *= 77)

For protocol 2 (and 3), sample size calculation including the estimated effects and known standard deviations in the pain tests revealed groups of 10 animals. To account for putative drop-outs, 11 animals were included in all groups. Similar to the procedures used in clinical studies, allocation to the respective treatment groups was randomized and observers were blinded with respect to the underlying treatment the animals received.

In order to identify an effective anti-hyperalgesic dose of intra-articularly injected NASHA (Durolane™ 20 mg/ml, Q-Med AB, Uppsala, Sweden), different volumes of NASHA (10, 30, 50, 75, and 100 μl, *n *= 11 per group) were injected into the left knee joint once. Then, behavioral tests indicating locomotor and pain-related behavior (see below) were performed on days 1, 7, 14, and 21 after treatment. Data were compared with those obtained from animals receiving a single treatment with saline according to NASHA treatment, or intraperitoneal injections of morphine (2.5 mg/kg; Sigma, Deisenhofen, Germany) on each testing day, approximately 30 minutes prior to bradykinin/PGE_2 _injection. Animals were randomized and group allocation was unblinded at the end of experiments, so except the morphine-treated animals, observers were unaware of the respective treatment.

The antinociceptive effect of each NASHA dose was calculated for each testing day using the mechanical thresholds (MT) from the injected knee (also see below):

Effects were logarithmically plotted against the NASHA dose used. Linear and sigmoid curves were fitted using a four parameter logistic function (Origin 8.1G, OriginLabs, USA).

#### Comparison between different hyaluronic acid preparations (protocol 3, *n *= 33)

The following clinically applied HA preparations were used: NASHA, Hylan GF20 (Synvisc™, Genzyme Biosurgery, Cambridge, MA, USA) and sodium hyaluronate (Hyalgan™, Fidia, Padua, Italy). As an injection volume of 50 μl proved to induce a significant antinociceptive effect (see results section), injection volumes of the remaining compounds were adapted according to clinical injection volumes in humans. For NASHA, this is 3 ml, for Hylan GF20 6 ml and for sodium hyaluronate 2 ml, resulting in rat intra-articular injection volumes of 50 μl, 100 μl, and 33 μl, respectively (*n *= 11 per group). Again, animals were randomized and unblinding was performed at the end of experiments. Similar to protocol 2, substances were injected intra-articularly once, and behavioral experiments were performed on days 1, 7, 14, 28, and 56. In order to quantify the antinociceptive effects of the three substances over time, areas under the curve (AUC) were calculated for saline and each of the HA preparations. The areas used for analyses were the integrals over the time points assessed. These were calculated using the mean of respective differences from the baseline value for each group for two consecutive time points when testing took place, for example days 1 and 7, multiplied with the number of days in this interval. The total area was obtained by adding the values from all intervals (1 to 7, 7 to 14, 14 to 28, and 28 to 56). The antinociceptive effect was then calculated as:

In this calculation, an antinociceptive effect of 0% means a reduction in thresholds/weight force to the same extent as saline-treated animals, while 100% would indicate a complete return to baseline values on all testing days.

### Behavioral tests

#### Assessment of mechanical pain-related behavior

Primary hyperalgesia at the site of the inflamed knee was assessed using a dynamometer (Correx, Berne, Switzerland) as described previously [41]. In brief, increasing pressure was applied to the lateral side of the knee joint at the level of the joint space until the animals attempted to escape or vocalized. The weight force to elicit this response was read out in grams. For each animal and testing day, this test was performed once. To prevent tissue damage, a cut-off value of 250 g was defined.

Pain-related guarding behavior of the inflamed hindpaw was assessed by quantification of weight bearing towards the non-inflamed hindlimb using an incapacitance tester (Linton Instrumentation, Norfolk, UK). Here, animals were placed in a plastic cage with both hindpaws resting on scales. After accommodation to the device when the animal was sitting calmly, the weight force resting on the two scales was obtained and averaged during three seconds and values from three consecutive measurements were averaged for every testing day. From these values, the relative weight (in %) resting on the inflamed hindlimb was calculated (weight on inflamed hindlimb × 100%/weight on the inflamed + the non-inflamed hindlimb) as described previously [42].

Secondary, hyperalgesia was assessed at sites remote from the inflamed joint: the paw and the contralateral knee joint. Mechanical secondary hyperalgesia at the contralateral knee joint was assessed according to the description given for the inflamed knee above. In addition, secondary mechanical hyperalgesia was obtained from the paw using a dynamic plantar aesthesiometer (Ugo Basile, Comerio, Italy) as previously described [43]. In brief, a blunt filament touches against the paw with increasing pressure (2.5 g/s, cut-off 50 g) until the animal withdraws, and the weight force needed to elicit this response is read out in grams. Measurements were taken in triplicate.

#### Locomotor behavior

To test for dynamic motor behavior and locomotor coordination, animals were tested on an accelerating RotoRod device (IITC Instruments, CA, USA). Animals were placed on a drum with 8 cm in diameter that started to rotate in an accelerating fashion, increasing from 4 to 40 rpm in 300 second. The speed at which the animal became unable to stay on the drum was obtained and used as readout parameter.

In addition, a guarding score was assessed as described previously [44]: 0: no guarding, 1: guarding of the hindlimb after a defined brief noxious compression of the knee, 2: visible limping during walking without previous pain stimulus, 3: no use of the hindlimb with the arthritic knee, 4: no movement at all (general morbidity).

### Statistical analyses

For statistical analyses, SPSS for windows (version 17.0) was used. First, data were tested for normal distribution applying Kolmogorov-Smirnov tests. For protocol 1, different doses were compared with baseline values using paired two-sided t-tests applying Bonferroni-Holm correction for multiple comparisons. For protocols 2 and 3, the measures obtained from different time points were compared between groups using repeated measures analysis of variances (ANOVAs) with the between-subjects factor *Group *(NASHA doses of 10, 30, 50, 75, and 100 μl for protocol 2; NASHA, Hylan GF20 and sodium hyaluronate for protocol 3) and the within-subjects factor *Time *(baseline, days 1, 7, 14, and 21 after initiation of treatment for protocol 2, and baseline, days 1, 7, 14, 28, and 56 for protocol 3). Antinociceptive effects over time (protocol 3) were compared between groups using one-way ANOVAs. *Post-hoc *t-tests were performed to describe differences between groups at different time points whenever ANOVAs revealed significant overall effects. For protocol 2, only injection volumes 10, 50, and 100 μl were compared in *post-hoc *tests in order to avoid multiple comparisons. F-values from multivariate tests are presented in the text, while *P *values from *post-hoc *t-tests are displayed in the figures and tables. Significance was accepted for *P *< 0.05.

## Results

### Validation of the bradykinin/PGE2 pain model (Protocol 1)

**Figure 1:**
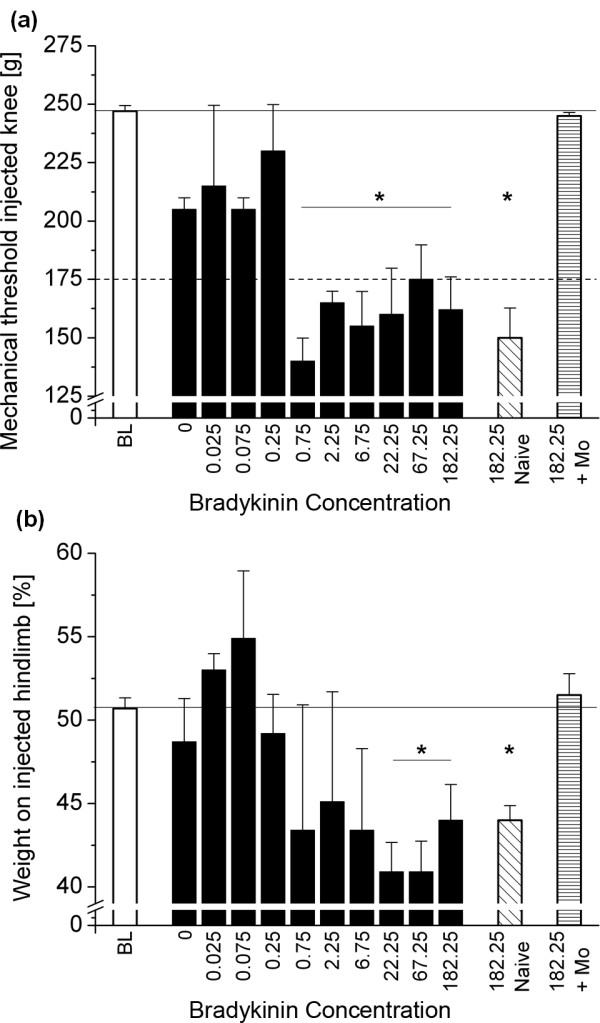
**Induction of transient pain by co-injection of PGE**_**2 **_**(0.5 μg) and bradykinin at different concentrations**. **(a) **Primary mechanical hyperalgesia as assessed by ascending pressure applied to the knee joint. Here, the desired drop in mechanical thresholds from baseline (BL) of more than 30% was obvious starting from 0.75 μg bradykinin in an escalating dose design (*n *= 4). For the chosen dose of 182.25 μg, this was verified in bradykinin-naïve animals (*n *= 4). Furthermore, the pain-related behavior induced by this concentration could be reversed by morphine (Mo; *n *= 4). **(b) **Weight force on the injected hindpaw (as percentage of total weight on both hindpaws). Here, a significant effect was obvious for concentrations of 22.25 μg and higher. Again, this effect could be verified in bradykinin-naïve animals and morphine administration prevented weight shifting. Data are presented as mean ± standard error of the mean. * *P *< 0.05 as obtained using t-tests applying Bonferroni-Holm correction. PGE_2, _prostaglandin E_2_.

Injection of 0.5 μg PGE_2 _together with different concentrations of bradykinin led to a decrease in mechanical thresholds. For doses up to 0.25 μg of bradykinin, this effect was smaller than the desired 30% reduction (corresponding to a weight force of 175 g in the mechanical threshold testing). Starting from 0.75 μg of bradykinin; however, a significant decrease below 175 g assessed 120 minutes after injection was obtained (Figure 1a). Injection concentrations of 0.25 μg of bradykinin or higher further induced transient licking of the injection side. In addition, concentrations between 0.25 and 2.25 μg caused limping upon defined noxious stimulation (according to a score of '1') for about 15 to 20 minutes, while concentrations of 6.75 μg and higher mainly caused visible limping without prior stimulation (according to a score of '2') for about the same time. Besides primary mechanical hyperalgesia, animals showed pronounced and statistically significant weight bearing starting from 22.25 μg bradykinin (Figure 1b).

As no adverse effects were observed up to a concentration of 182.25 μg, and as at this concentration all parameters indicating pain, that is a decrease in thresholds, a significant weight shifting, licking, and limping could be observed reliably, this dose was chosen and used in an additional four animals that had not received any other bradykinin/PGE_2 _injection before in order to verify the effect in naïve animals (Figure 1).

Application of morphine 30 minutes prior to bradykinin/PGE_2 _injection completely abolished the hypernociceptive effect as assessed using mechanical thresholds and weight bearing, thereby confirming that the measures obtained indeed indicate pain (Figure 1).

### Dose response relationship for NASHA regarding pain-related behavior (protocol 2)

**Figure 2:**
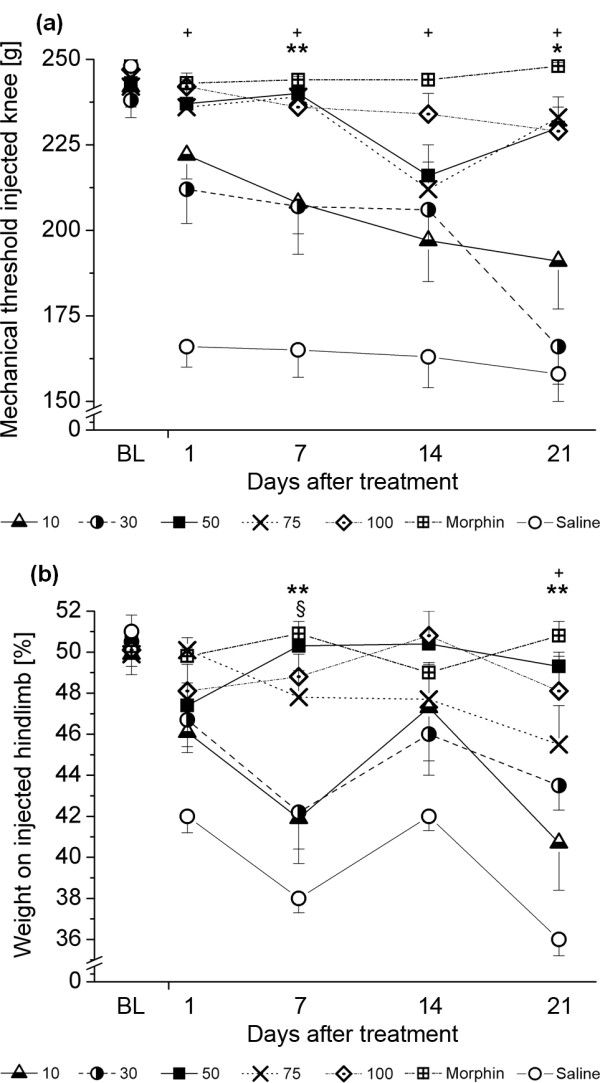
**Time course of the antinociceptive effects upon a single intra-articular injection of different NASHA doses**. **(a) **Primary mechanical hyperalgesia at the knee joint as assessed by measuring the mechanical threshold upon ascending pressure applied to the knee joint. NASHA doses were 10, 30, 50, 75, and 100 μl (each *n *= 11, except 30 μl, *n *= 10). Here, the lower doses used, 10 and 30 μl injection volumes, showed a linear decrease, while the higher doses did not significantly differ from baseline (BL) levels. **(b) **Weight force on the injected hindpaw (as percentage of total weight on both hindpaws). Same doses as in a. The effects were similar, yet less clear-cut than those obtained from mechanical thresholds, but verified a shorter-lasting and smaller efficacy of the lower doses. Data are presented as mean ± standard error of the mean. + comparison between NASHA 10 and NASHA 100; * comparison between NASHA 10 and NASHA 50; § comparison between NASHA 50 and NASHA 100. One symbol: *P *< 0.05; two symbols: *P *< 0.01 as obtained from descriptive t-tests following repeated measures analysis of variances.

Repeated measures ANOVAs showed significant *Time × Group *interactions for primary mechanical hyperalgesia assessed as mechanical thresholds at the injected knee joint (F(16,141) = 1.947; *P *= 0.021) and for weight bearing as obtained from incapacitance testing (F(16,141) = 1.798; *P *= 0.042). Results from *post-hoc *t-tests are displayed in Figure 2. Here, the lower application volumes of 10 and 30 μl showed a rather linear decrease in MTs during the observation period of 21 days, while the higher volumes administered remained close to baseline levels and morphine treatment (Figure 2a). For weight bearing, a similar effect was observed, with more pain-related weight shifting in animals receiving the low doses (Figure 2b). No differences were observed in secondary mechanical hyperalgesia (data not shown) or in locomotor coordination (F = 1.174; *P *= 0.296).

**Figure 3:**
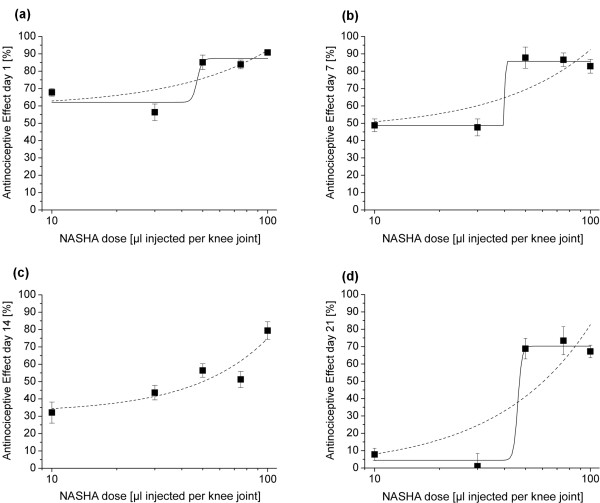
**Dose-response relation for NASHA regarding antinociceptive effects on different days**. **(a to d) **Increase in thresholds in relation to saline (0%) and morphine (100%) on days 1, 7, 14, and 21 after injection. Overall, only the higher doses (50, 75, and 100 μl, each *n *= 11) show an antinociceptive effect of more than 50% beyond day 1, but not 10 and 30 μl (*n *= 11 and *n *= 10, respectively). Fitting of linear and sigmoid curves (only linear fitting was possible for day 14) revealed no clear-cut relation, but apparently a certain threshold dose is needed to obtain antinociceptive effects. Data are presented as mean ± standard error of the mean.

For each testing day, antinociceptive effects were plotted against the administered volume of NASHA (Figure 3). From this, it becomes obvious that all concentrations used show an antinociceptive effect of more than 50% on day 1 (Figure 3a), but that the low concentrations used (10 μl and 30 μl) show a decline in efficacy over time (Figures 3b to 3d), while the higher injection volumes remain rather stable at effects above 50%.

Fitting of linear and sigmoid curves using logarithmic interpolation showed that neither of the two relations sufficiently described the dose-response relation, but that rather a certain amount of NASHA needs to be administered in order to achieve an antinociceptive effect. This threshold dose lies between 30 and 50 μl injection volume. For day 14, only a linear fit could be calculated (Figure 3c).

### Comparison between clinically used HA formulations (protocol 3)

**Figure 4:**
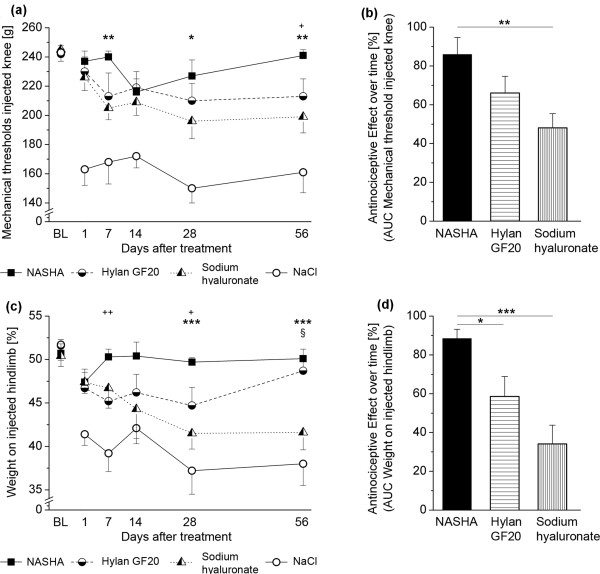
**Antinociceptive effects of NASHA, Hylan GF20 and sodium hyaluronate during an observation period of 56 days**. **(a) **Primary mechanical hyperalgesia as assessed by ascending pressure applied to the knee joint, after injection of NASHA (50 μl, *n *= 11), Hylan GF20 (100 μl, *n *= 9), and sodium hyaluronate (33 μl, *n *= 11). Although saline-treated animals showed a dramatic drop in mechanical thresholds from day 1, all hyaluronic acid compounds showed antinociceptive properties. These were most pronounced for NASHA and Hylan GF20, which were superior to sodium hyaluronate, particularly in the later stages. **(b) **When calculating the area under the curve (AUC) in order to quantify the antinociceptive effects of these substances (baseline curve - saline curve), NASHA showed a significantly stronger effect than sodium hyaluronate, whereas only a trend was observed in comparison with Hylan GF20. **(c) **Weight force on the injected hindpaw (as percentage of total weight on both hindpaws). Same dosing as in a. Here, a similar pattern was obvious, with particularly sodium hyaluronate losing efficacy from day 7 after injection, while NASHA, and to a lesser degree Hylan GF20, maintained weight-bearing behavior close to baseline levels. **(d) **Calculation of the respective antinociceptive effects for this parameter showed significant differences between NASHA and Hylan GF20 as well as between NASHA and sodium hyaluronate. Data are presented as mean ± standard error of the mean. **(a and c) **+ comparison between NASHA and Hylan GF20. * comparison between NASHA and sodium hyaluronate. § comparison between Hylan GF20 and sodium hyaluronate. One symbol: *P *< 0.05; two symbols: *P *< 0.01 as obtained from descriptive t-tests following repeated measures analysis of variances (ANOVAs). (b and d) * *P *< 0.05; ** *P *< 0.01 as obtained from descriptive t-tests following one-way ANOVAs.

As an injection volume of 50 μl proved to induce a significant antinociceptive effect (see results section), injection volumes of the remaining compounds were adapted according to clinical injection volumes in humans. For NASHA, this is 3 ml, for Hylan GF20 6 ml and for sodium hyaluronate 2 ml, resulting in rat intra-articular injection volumes of 50, 100, and 33 μl, respectively (*n *= 11 per group). Repeated measures ANOVAs showed significant *Time × Group *interactions for primary mechanical hyperalgesia (F(15,94) = 3.550; *P *< 0.001) and weight bearing (F(15,94) = 2.646; *P *= 0.002). In particular, MTs at the injected knee were significantly higher in NASHA-treated animals than in sodium hyaluronate-treated animals on days 7 and 56 after injection (Figure 4a). The antinociceptive effect over time using AUC analyses for this parameter was significantly different between groups (F = 5.630; *P *= 0.009, Figure 4b). For weight bearing, animals treated with NASHA showed the mildest shift of weight, particularly on the late observation days (Figure 4c). Here, the overall antinociceptive effects showed an even stronger differentiation between groups (F = 11.178; *P *< 0.001) with NASHA being slightly more effective than Hylan GF20 and strongly more antinociceptive than sodium hyaluronate (Figure 4d).

Secondary mechanical hyperalgesia as assessed at the contralateral knee (F = 0.837; *P *= 0.634) or at the ipsi- and contralateral paws (F = 0.993; *P *= 0.469 and F = 0.789; *P *= 0.693, respectively) was not different between treatment groups. Furthermore, there were no differences in locomotor coordination as assessed using the RotoRod device (F = 0.604; *P *= 0.865).

## Discussion

In the present study, we were able to validate the antinociceptive effects of HA preparations in a highly reproducible animal pain model using repeated intra-articular injections of bradykinin and PGE_2_. In this model we established a dose-response relation for the HA formulation of NASHA, showing that smaller injection volumes provided a weaker and shorter lasting effect than higher volumes. In addition, using clinically administered injection volumes in humans as a reference, NASHA was compared with different HA formulations, that is Hylan GF20 and sodium hyaluronate, with regards to pain-related and locomotor behavior. Overall, in the first days after injection all the HA preparations showed antinociceptive effects over that of intra-articular injection of saline, negative control. However, particularly in the long-term range the effectiveness of the tested HA products differed, with NASHA having the strongest antinociceptive action, followed by Hylan GF20, then sodium hyaluronate, under the conditions of these experiments.

### Use of the bradykinin/PGE_2 _model for the study of HA effects (protocol 1)

Intra-articular injection of bradykinin and PGE_2 _led to a reproducible, repeatedly applicable and significant change in pain-related behavior as assessed employing different methods (see effects of saline and morphine injections). Although in principle, such models, mainly using bradykinin alone, have been used before [25,26], we could now validate this model for the assessment of long-lasting antinociceptive effects of a single injection of HA preparations in individual animals. In previous studies the antinociceptive effects of a HA preparation were assessed for a maximum of 96 hours, and bradykinin was only injected once [25]. It is particularly worth mentioning that repeated applications of bradykinin and PGE_2 _did not induce tachyphylaxia in this design, and that the anticipated effect of a reduction in mechanical thresholds of more than 30% was present on all testing days up to week 7.

The employed pain model does not reflect all aspects of clinical OA or other joint diseases. It should be noted, however, that there is no consensus in pain research which model is most suitable to study OA pain. This also reflects the clinical situation. So far the pain mechanisms of OA are not well understood. However, the model provides a rather reliable, fast, and efficient way to address the antinociceptive effects of single injections of HA preparations in a long-term design *per se*. It may mimic pain conditions at a stage of OA, which evokes episodically moderate pain and does not require the use of strong analgesics or systemic pain treatment. An advantage is that the effects observed herein can be attributed directly to the antinociceptive effects of HA rather than to disease modification. Finally, the protocol of repetitive induction of short-lasting pain states limits suffering of experimental animals. Pain is totally avoided when nerve fibres are recorded in anesthetized animals [21-24], but in these experiments measurements are usually restricted to one day (or time point) only.

### Dose-response relationship of NASHA (protocol 2)

In order to obtain quantitative data on the dose-response relation with regards to pain-related behavior, five different doses of NASHA were administered. As NASHA consists of a fixed chemical structure, the dosing is established by injecting different volumes into the knee joint. When looking at previous studies and animal models employing knee joint injections, a volume of 50 μl appeared to be mostly used [25,26,39,45,46] and - considering joint volumes between species - comparable with respective injection volumes in humans. From this, two larger and two smaller doses were chosen. Dose-dependently, these injection volumes reduced pain-related behavior, as reflected in an attenuated decrease of MTs upon bradykinin/PGE_2 _injections and in a normalization of the weight shift seen in saline-treated control animals. The antinociceptive effects of doses of 50 to 100 μl were similar as those obtained with morphine. Importantly, doses of 50 μl or more had an antinociceptive effect throughout the observation period of 56 days.

The original aim of this design was to establish a logarithmic curve, from which ED50 values might be obtained. From the data, however, no clear-cut sigmoid or linear relation could be established. Rather, there appears to be a certain threshold dose or volume that needs to be injected in order to achieve therapeutic effects, which, in our study, lies between 30 and 50 μl. Only the higher doses yielded persistent effects up to 56 days. An additional increase of injection volumes did not result in dramatically stronger or longer-lasting effects, at least considering the observation period in this study.

### Putative mechanisms of hyaluronic acid effects

The mechanisms underlying the beneficial effects of HA in OA pain/degenerative pain are not completely understood to date. As putative modes of action, the viscous properties of the substances have been discussed, acting as a mechanical protection for the joint. Furthermore, due to the texture of the respective preparations, HA might be capable of covering sensory endings that can then no longer be sensitized by inflammatory mediators [24]. Alternatively, these mediators which also include those used in our model to induce the acute pain states, that is bradykinin and PGE_2_, might be entrapped in the viscous compound, thereby being unable to reach the respective receptors in sufficient concentration. Indeed, some preliminary work has indicated that NASHA can bind and hold bradykinin possibly through electrostatic interaction (data not shown). Besides that, HA represents, under healthy conditions, the major component of synovial fluid and fulfils important trophic-metabolic functions [47-49]. Irrespective of the exact mechanism, recordings from afferent nociceptive fibers in anesthetized animals showed reduced excitability upon intra-articular treatment with HA [21-24]. Ultimately, only deeper insights in of these mechanisms will allow the understanding of the threshold effect described here.

### Comparison between hyaluronic acid preparations (protocol 3)

For comparison of the antinociceptive effects of different compounds we adapted the volumes of the preparations according to clinical injection volumes in humans (see Results). Furthermore, we took into account that for the injection of the rat knee joint a volume of 50 μl is most suitable (and has hence been routinely used for studies, see above) whereas an OA human knee volume was estimated to be more than 3 ml [50]. Thus, with 50 μl NASHA we achieved a similar 1:1 ratio between the injected volume and the physiological joint space as when 3 ml NASHA (the usually applied dose) are injected into a human knee joint. Under these conditions, NASHA showed the strongest antinociceptive effects, followed by Hylan GF20, while sodium hyaluronate - despite showing good efficacy in the very early testing days - was less potent, particularly in the late stages of the observation period. For almost all mechanisms discussed for the effects of HA in degenerative joint disease mentioned above, it appears to be of major importance for how long the substance can actually remain in the joint cavity before being washed out. In that respect, NASHA and Hylan GF20, the two longer-lasting and more effective substances, have in common that they are cross-linked and thereby less likely to be cleared from the joint as rapidly as the non-cross-linked sodium hyaluronate. The even stronger effect of NASHA might therefore be caused by the chemical structure of gel beads that release the HA more slowly, and the gel nature of this preparation preventing an early washout. In addition, different half life times of the compounds were reported. For unmodified hyaluronan like sodium hyaluronate, this is 12 to 24 hours [51], for Hylan GF20 approximately up to 8.8 days [52], and for NASHA 28 to 32 days [33,34], thereby possibly adding to the explanation of the longer-lasting effect of the latter.

### Limitations and advantages

The comparison between substances was performed by establishing a dose-response relation for NASHA, and calculating the injection volumes of the compared substances according to clinically used amounts. Therefore, the absolute amounts of HA injected differ between substances. Particularly, the least effective substance, that is sodium hyaluronate, was injected in a rather small volume only (corresponding, however, to the clinically injected volume, see above), thereby putatively confounding our results. Furthermore, sodium hyaluronate is an unmodified hyaluronan, which has the shortest half-life time (see above) and is rapidly removed from the joint space and therefore needs to be re-injected three to five time in weekly intervals. This might also explain the rather weak effect of this substance in the model used. Pointing in the same direction, in recordings from nerve fibers of joints, sodium hyaluronate did not reduce the discharges of the nerve fibres whereas in the same experimental setting Hylan GF20 reduced the impulse frequency [22].

As different compounds were compared in the present study and in order to reduce any bias due to expectations, we applied a group size estimation, randomization, and blinding process which is usually only used in clinical studies, thereby increasing internal validity [53] and adding value to the results shown here.

## Conclusions

The injection of HA preparations into OA joints is often used to treat OA pain. However, the assessment of the antinociceptive effects of HA preparations solely from human studies is difficult for several reasons, namely the strong placebo effect upon intra-articular injection, the long duration of the observation period (eventually with disease progression), the difficulty to test different doses of one compound, and the difficulty to compare different HA preparations. The present study shows that long-term antinociceptive effects of HA preparations can be assessed in an animal model of joint pain based on the repeated intra-articular injection of bradykinin and PGE_2_. Injection of these compounds (during short anesthesia) causes a transient short-lasting joint pain state, and upon repeated injections this pain induction is reproducible over weeks. In this pain model, a single injection of 50 μl of NASHA and higher into the knee joint led to a normalization of pain-related behavior close to baseline levels during an observation period of seven weeks. When injection volumes in rat knee joints were adapted to clinical injection volumes in humans, NASHA showed slightly better antinociceptive effects than Hylan GF20, and both substances were superior to sodium hyaluronate.

Overall, this study has demonstrated that all tested HA preparations are effective in providing pain relief when injected into the joint. Remarkably, NASHA and Hylan GF20 (as Synvisc™ One) are the only products that are currently available as single injections. In the pain model employed in the present study, NASHA provided the best prolonged antinociceptive effect upon a single intra-articular injection.

## Abbreviations

ANOVA: analysis of variation; AUC: area under the curve; HA: hyaluronic acid; MT: mechanical threshold; NASHA: stabilized hyaluronic acid from a non-animal source; NSAID: nonsteroidal anti-inflammatory drug; OA: osteoarthritis; PGE_2_, prostaglandin E_2_.

## Competing interests

AH is employed by Smith & Nephew and holds stocks and shares in Smith & Nephew. The Institute of Physiology, University Hospital Jena, was contracted by Smith & Nephew to conduct this study. Test substances were supplied and the Institute received funding from the company. However, no salary was paid to any of the authors employed by the University Hospital Jena.

## Authors' contributions

MKB designed the study, took responsibility for animal healthcare, carried out part of the experiments, took care of the unblinding procedure, performed the statistical analysis, interpreted the data and wrote the manuscript. DK carried out parts of the experiments and statistical analysis. AH initiated the study, provided knowledge on the HA preparations and their clinical use, designed the study, and contributed to the manuscript. HGS designed the study, interpreted the data, and wrote the manuscript. All authors read and approved the final manuscript.
